# The impact of nursing practice environments on patient safety culture in primary health care: a scoping review protocol

**DOI:** 10.3399/BJGPO.2023.0032

**Published:** 2023-07-12

**Authors:** Soraia Cristina de Abreu Pereira, Olga Maria Pimenta Lopes Ribeiro, Cintia Silva Fassarella, Eduardo José Ferreira Santos

**Affiliations:** 1 Abel Salazar Biomedical Sciences Institute, University of Porto, Portugal and North Region Health Administration, Porto, Portugal; 2 Nursing School of Porto and Center for Health Technology and Services Research Faculty of Medicine, Porto University, Porto, Portugal; 3 Faculty of Nursing of the State University of Rio de Janeiro, University of Rio de Janeiro, Brazil; 4 Nursing School of Coimbra, Health Sciences Research Unit: Nursing and Portugal Centre for Evidence Based Practice (PCEBP): a JBI Centre of Excellence, Coimbra, Portugal

**Keywords:** environment, nursing, patient safety, primary health care, general practice

## Abstract

**Background:**

Patient safety is a key priority for healthcare organisations. It impacts directly on patient health and wellbeing. The increasing complexity of current healthcare settings, which are associated with high work demands and increasingly stressful professional practice environments, contributes to an increased likelihood of errors and adverse events. Primary health care, given its comprehensiveness of care, makes up a large proportion of the care delivered to the population.

**Aim:**

To map the knowledge about the impact that nursing practice environments have on safety culture in the primary healthcare setting. This knowledge is essential for a more effective and appropriate understanding of this phenomenon and to enable the definition of strategies that can promote the provision of safer care to the population.

**Design & setting:**

A scoping review will be conducted based on the method proposed by the JBI, and the Preferred Reporting Items for Systematic Reviews and Meta-Analyses extension for Scoping Reviews (PRISMA-ScR) will be used.

**Method:**

Study selection, data extraction, and synthesis will be performed by two independent reviewers. Based on the Population (or participants), Concept, and Context (PCC) framework, this scoping review will consider studies that address nurses' practice environment and patient safety culture in primary health care. The review will consider all studies, published or unpublished, from 2002 to the present.

**Conclusion:**

The results from this scoping review are expected to provide an overview of the importance of the nursing practice environments on patient safety culture, which will be crucial to define an appropriate range of strategies to promote the delivery of the safest health care to the population.

## How this fits in

Primary health care is the main gateway to health services, and is the setting in which a large proportion of the care delivered to the population is concentrated. The results of this scoping review will be used to understand the impact that nursing practice environments have on patient safety culture in the specific context of primary health care. There has been little research in this setting related to issues of patient safety and nursing practice environments. The knowledge that this review may bring is essential to understand more effectively the relationship between nursing practice environments and patient safety, which will be crucial to define appropriate strategies that promote the safest care delivery to the population.

## Introduction

Patient safety is a key area of focus for healthcare organisations. It impacts directly on patient health and wellbeing, and is closely connected with economic costs.^1^ The increasing complexity of current healthcare settings, associated with high work demands and increasingly stressful professional practice environments, contributes to an increased likelihood of errors and adverse events.^2–7^ According to World Health Organization (WHO) data, the provision of unsafe care is responsible for the annual global loss of 64 million disability-adjusted life years, and is one of the top 10 causes of disability and death worldwide.^8,9^ Aware of the scale of the global patient safety issue, in 2004 the WHO created the World Alliance for Patient Safety, with the goal to establish concepts while developing guidelines and recommendations to reduce risks and adverse events in health systems.^2,10^

Patient safety can be defined as *'the process of amelioration, avoidance, and prevention of adverse injuries or outcomes that arise as a result of the healthcare process'.* ^11^ Safety culture is understood as *'the product of individual and group values, attitudes, perceptual skills, and behavioural models',* ^12^ and it is essential that there is trust and open communication within organisations so that professionals collaborate in identifying problematic situations and unsafe environments.^6,13,14^

The 2004 Institute of Medicine (IOM) report, *Keeping Patients Safe: Transforming the Work Environment of Nurses,* ^15^ validated this relationship and made important reflections, highlighting the importance of nurses' involvement in organisational management; the nurse-to-patient ratio^16^; the need for active nursing leadership; and the need for constructive work environments to promote patient safety.^15,17^ In 2020, the WHO also highlighted the need to link patient safety and professional practice environments, and established the theme 'Safe health workers, safe patients' on World Patient Safety Day that year.^18^

The work environment, and specifically the practice environment of nurses, is indeed an important variable related to patient safety, and this relationship has been recognised in several studies conducted in the past years.^1,17,19–21^ In 2021, Jarrar and colleagues also concluded that healthy nursing practice environments were related not only to improved quality of care, but also to an evident reduction of patient harm.^19^ Aspects such as heavy workloads; lack of staff and resources; miscommunication; and lack of staff involvement in organisational policies and decision making negatively affect the psychological and physical health of nurses,^17^ increase the likelihood of adverse events occurring,^19^ and have been associated with poorer patient safety ratings by nurses.^1^

The factors highlighted in recent studies as being the most important for improving the quality and safety of care include staff recruitment; human and material resources adequacy; participation in health organisation decisions; promotion and enhancement of nurses; communication^17,19^; cooperation between nurses and other professionals; and support received by nurse managers.^1,20^

Therefore, the following points should be focused on to improve care delivery and make care safer: nurses should be involved in the decisions of healthcare organisations; they should be provided with appropriate training in quality development programmes; they should be given leadership support; and they should be involved in interdisciplinary relationships.^17,21,22^ Nurse managers play a key role in promoting positive and safer practice environments by enhancing nurse involvement and participation; encouraging nurses to adopt evidence-based practices and clinical research; and providing staff training related to safer and higher quality care.^19^

Good nursing practice environments have a positive correlation not only with patient safety culture, as evidenced in the aforementioned recent studies, but also they are important for increasing nurses' job satisfaction, as well as their involvement in institutions and in the profession itself, decreasing the risk of burnout and, consequently, resulting in the provision of higher quality care.^21^

Patient safety has been widely studied in recent years in hospital settings, although primary health care has received less attention; however, adverse events occur in this setting not only owing to high workloads, but also because of limitations in infrastructure and procedures.^6,22^ Safe primary health care is a global priority for the WHO, not only because the patient is not under continuous supervision, but also because of the difficulty in sometimes identifying safety-related incidents of care.^5,23,24^

Whatever the level of health care, patient safety is an unquestioned priority and the importance and need for risk-free, effective, and efficient health care is well recognised. Primary health care, given its comprehensiveness of care — which includes health promotion, disease prevention, diagnosis, treatment, and rehabilitation interventions^10,25^ — represents a concentration of a large proportion of the care delivered to the population.^26^ Therefore, it is essential to understand the impact that nursing practice environments have on patient safety culture in the specific context of primary health care. This knowledge is essential for a more effective and appropriate understanding of this phenomenon, and to enable the definition of strategies that can promote the provision of safer care to the population.

A preliminary search of JBI Evidence Synthesis, the Cochrane Database of Systematic Reviews, PROSPERO, Open Science Framework (OSF), and MEDLINE was conducted and two scoping reviews were identified as being relevant. The first aimed to map evidence about the nursing practice environment in primary health care, but did not address the concept of patient safety, which is central to this study;^7^ the second identified the patient safety challenges reported by health professionals in primary health care, but did not address the issue of nursing practice environments.^27^ Besides the aforementioned reviews, no other current or in-progress scoping reviews or systematic reviews on the topic were identified and the primary evidence is poorly described. Mapping all the knowledge about the impact that nursing practice environments have on safety culture is, therefore, a relevant and informative undertaking.

The objective of this scoping review is to map the knowledge about the impact that nursing practice environments have on safety culture in the primary healthcare settings. The review question is as follows: what impact do nursing practice environments have on patient safety culture in primary health care?

## Method

The proposed scoping review will be conducted in accordance with the JBI methodology for scoping reviews^28^ and the PRISMA-ScR.^29^ The current protocol followed the Preferred Reporting Items for Systematic Reviews and Meta-Analyses Protocols (PRISMA-P).^30^

### Eligibility criteria

The eligibility criteria were developed according to the PCC framework.^31^ Regarding the participants, the review will consider studies that include nurses in any field of action. The concept under study in this review is evidence mapping related to patient safety culture and nurses' practice environments.

Patient safety is defined by the WHO as *'the reduction of risk of unnecessary harm associated with health care to an acceptable minimum'* ^32^ and a patient safety incident is *'an event or circumstance which could have resulted, or did result, in unnecessary harm to a patient'.* ^32^ The safety culture is considered the *'product of individual and group values, attitudes, perceptions, competencies, and patterns of behaviour that determine the commitment to, and the style and proficiency of, an organisation’s health and safety management'.* ^32^

Nursing work environment can be defined as *'the set of characteristics of the work context that facilitate or constrain the professional nursing practice'*.^33^ For the International Council of Nurses (ICN) a favourable work environment is characterised by having a safe work environment; innovative policies focused on recruiting and retaining professionals; adequate remuneration for professionals; sufficient recognition programmes; and material resources.^34^

Studies that include and relate to these two concepts — patient safety culture and nurses' practice environments — with the definitions mentioned, will be considered in this review. Studies that guide or describe nurses' practice environments if they also consider suggestions related to patient safety or patient safety culture will also be included in this scoping review.

In terms of the context, this scoping review will include studies conducted in primary care settings or primary healthcare organisations regardless of the country of origin or sociocultural environment, considering the authors' interest in mapping the existing evidence. Primary care is organised in different ways in different countries, with each taking its own approach. This diversity of interpretations and implementations in different countries motivated the WHO and United Nations Children’s Fund (UNICEF) to join efforts to develop a clear and simple definition that would facilitate its interpretation.^35^ Thus, primary health care can be defined as *'a whole-of-society approach to health that aims at ensuring the highest possible level of health and wellbeing and their equitable distribution by focusing on people’s needs and as early as possible along the continuum from health promotion and disease prevention to treatment, rehabilitation and palliative care, and as close as feasible to people’s everyday environment'.* ^35^ Whereas in some countries primary health care operates based on a strong, nationwide healthcare system, in other countries primary health care consists of small services with few staff and resources, and in some cases these services are provided by private, independent healthcare institutions.^33^ Therefore, this review will consider studies conducted in primary care settings, primary healthcare organisations or centres, and community services or centres.

### Search strategy

There will be a three-step search strategy in this review. First, an initial limited search of MEDLINE (PubMed) and CINAHL (Cumulative Index to Nursing and Allied Health Literature; EBSCO) was undertaken to identify articles on the topic. The words contained in the titles and abstracts of relevant articles, and the index terms used to describe the articles were used to develop a full search strategy (Supplementary Table S1). A second search using all the identified keywords and index terms will be undertaken across the following databases: MEDLINE (PubMed); CINAHL (EBSCO); and Embase (Elsevier). When searching for unpublished studies, the following databases will be searched: Repositório Científico de Acesso Aberto de Portugal (RCAAP); WHO; Agency for Health Research and Quality; WorldCat; and ProQuest Dissertations and Theses Global. In the third step, the reference lists of articles included in the review will be screened for additional articles.

The review will consider literature published or unpublished in any language. Articles published from 2002 to the present will be included, as in January 2002 the WHO Executive Board discussed the topic of patient safety extensively, and since then many member states have taken initiatives on patient safety within their own healthcare systems, becoming a very important milestone in patient safety.

This scoping review will consider quantitative studies (for example, experimental, quasi-experimental, prospective, and retrospective cohort, case-control, cross-sectional), observational studies (for example, case series, individual case reports, descriptive cross-sectional studies), qualitative studies (for example, phenomenology, ethnography, qualitative description), mixed-methods studies, systematic reviews, opinion articles, and unpublished studies. This will allow a greater sensitivity in the search, which is desirable for this scoping review.

### Study selection

Subsequently to the search, all identified records will be collated and uploaded into EndNote (version X9.3.3) and duplicates will be removed. The titles and abstracts will be reviewed by two independent reviewers to assess the eligibility of the studies against the initially defined inclusion criteria. A pilot screening process will be conducted independently by both reviewers on an initial 25 titles and abstracts. The results of the screening will then be compared and discussed, while allowing changes to the eligibility criteria to ensure that both reviewers agree, if required. This pilot process will continue until at least 75% agreement between the reviewers is reached.^31^ The full text of articles that meet or potentially meet the inclusion criteria will be reviewed. The full text of selected citations will be assessed in detail against the inclusion criteria by two independent reviewers. Any disagreements between the reviewers at each stage of the selection process will be resolved through a constructive discussion or by referring to a third reviewer. Citations from eligible studies retrieved in full will be imported into Rayyan. Any reasons for exclusion of full-text papers that do not meet the inclusion criteria will be recorded and reported in the scoping review. The results of the search will be reported in full in the final scoping review and presented in a PRISMA flow diagram.^36^

### Data extraction

Data will be extracted from selected studies included in the scoping review by two independent reviewers using a data extraction tool developed by the reviewers. The data extracted will include specific details about the concept, context and study methods, and objectives. The draft data extraction tool will be modified and revised as necessary during the process of extracting data from each included paper. Modifications will be detailed in the full scoping review. Once again, any disagreements that may arise between the reviewers will be resolved by a third reviewer. If necessary, the authors of the included articles will be contacted for further information or data clarification.

## Discussion

This scoping review aims to map the knowledge about the impact that nursing practice environments have on safety culture in primary healthcare settings. A possible implication of this study for practice and research is that it may allow the definition of appropriate strategies to implement interventions that, through the promotion of more positive and healthier nursing practice environments, can make the care provided safer.

It is believed that this scoping review will add evidence to clarify how patient safety can be influenced by nursing practice environments in the primary healthcare setting. Optimising nursing practice environments could enhance the patient safety culture in primary health care, by making care safer and reducing adverse events, which will have an undeniable impact on population health. It is hoped that this synthesised knowledge can bring important contributions to practice and research, developing new lines of enquiry, and influencing professionals and managers in decisionmaking.

## Trial registration number

This protocol is registered with the Open Science Framework https://doi.org/10.17605/OSF.IO/JG3HE.

## Data

All data relevant to this review are included in the article or provided as supplementary information.
